# How do animals weigh conflicting information about reward sources over time? Comparing dynamic averaging models

**DOI:** 10.1007/s10071-024-01840-2

**Published:** 2024-03-02

**Authors:** Jack Van Allsburg, Timothy A. Shahan

**Affiliations:** Department of Psychology, Utah State University, Logan, Utah USA

**Keywords:** Dynamic averaging, Spontaneous recovery, Choice, Temporal weighting rule, Concurrent schedules

## Abstract

Optimal foraging theory suggests that animals make decisions which maximize their food intake per unit time when foraging, but the mechanisms animals use to track the value of behavioral alternatives and choose between them remain unclear. Several models for how animals integrate past experience have been suggested. However, these models make differential predictions for the occurrence of spontaneous recovery of choice: a behavioral phenomenon in which a hiatus from the experimental environment results in animals reverting to a behavioral allocation consistent with a reward distribution from the more distant past, rather than one consistent with their most recently experienced distribution. To explore this phenomenon and compare these models, three free-operant experiments with rats were conducted using a serial reversal design. In Phase 1, two responses (A and B) were baited with pellets on concurrent variable interval schedules, favoring option A. In Phase 2, lever baiting was reversed to favor option B. Rats then entered a delay period, where they were maintained at weight in their home cages and no experimental sessions took place. Following this delay, preference was assessed using initial responding in test sessions where levers were presented, but not baited. Models were compared in performance, including an exponentially weighted moving average, the Temporal Weighting Rule, and variants of these models. While the data provided strong evidence of spontaneous recovery of choice, the form and extent of recovery was inconsistent with the models under investigation. Potential interpretations are discussed in relation to both the decision rule and valuation functions employed.

## Introduction

To forage effectively in highly variable natural environments, animals must make predictions to inform their behavior. High-quality predictions enable animals to make decisions that maximize food intake over time, a strategy entailed by optimal foraging theory (review in Pyke 1984). However, many factors relevant to these predictions are in constant flux—including weather, food availability, threats of predation or competition, and more. Consequently, animals must employ policies for integrating information that is inherently incomplete (as only a subset of factors can be tracked; McNamara and Houston 1980) and time-sensitive (as information ages, it is increasingly likely to no longer reflect current conditions; McNamara and Houston 1987). Relevant factors also change at considerably different rates—food availability may vary by the season, but predation risk could vary by the minute—meaning that animals must track both persistent and transitory trends to make optimal decisions. Models of valuation in foraging, therefore, have aimed to determine how animals balance short-term maximization and long-term maximization (reflecting current and historical conditions, respectively; Dow and Lea 1987).

As a general principle in this compromise, empirical evidence shows animals place more weight on recent experiences (Cowie 1977; reviewed in Stephens and Dunlap 2017), following from the notion that recent experience will more accurately describe current conditions and enable more optimal decisions. The earliest approach to modeling how animals track the value of foraging options over time (Cowie 1977) reflects this principle: the model uses an unweighted average of events within a specific “memory window” of time, excluding any older experience from this average.

The memory window’s arbitrary “cutoff” was revised through models based on an exponentially weighted moving average (EWMA, pronounced “yuma”) (Killeen 1981). To calculate value (*V*) for a given option at the current moment (*V*_*n*_) using a EWMA (Eq. 1), animals assign some amount of weight, determined by a free parameter* β*, to rewards received in their current experience (*q*_*n*_) and assign the remainder of weight (1 − *β*) to their past experience (*V*_*n-1*_, the valuation from the model’s previous update). Past experiences are therefore exponentially discounted in weight each time the model is recursively incremented.

1 $$V_{n} = \beta q_{n} + \left( {1 - \beta } \right)V_{{n - 1}}$$

EWMA-based models have been adopted widely enough in behavioral ecology, foraging, and decision-making to be termed the “common model” of dynamic averaging (Lea and Dow 1984). For example, EWMAs are employed in a variety of choice and dynamic averaging models (including Harley 1981; Myerson and Miezin 1980; Navarro et. al 2016; and others). However, models with learning rate parameters that integrate past experience with a *functionally* exponential decay are perhaps even more prevalent, including models based on the linear operator model (Bush and Mosteller 1951), such as the Rescorla–Wagner model of classical conditioning (1972), influencing widespread adoption of this approach in reinforcement learning (reviewed in Katahira 2015), computational neuroscience (Iigaya et al. 2019; Saito et al. 2014; reviewed in Niv 2009), and artificial intelligence (Su and Hsu 2004; Zhang et al 2013).

As an alternative to this approach, the temporal weighting rule (TWR; Devenport and Devenport 1993; 1994) treats past experience in a different way. According to the TWR, animals hold a record of experiences with an alternative (or patch) in memory, including the reinforcement received during each experience and how long ago each occurred. To calculate the value (*V*) of a given alternative at a given moment, reinforcement obtained during each experience (*q*_*x*_) is multiplied by that experience’s unique weight (*w*_*x*_) as of the current moment, then summed (Eq. 2):

2 $${\text{V}}= {\sum }_{x}{w}_{x}{q}_{x}$$

The weight for each experience (*W*_*x*_) is calculated with Eq. 3. The numerator calculates the recency of the experience as the reciprocal of *t*_*x*_ (the time from that experience to the present), while the denominator sums the recencies for all *n*_*k*_ experiences with the alternative.

3 $$W_{x} = \frac{{{1 \mathord{\left/ {\vphantom {1 {t_{x} }}} \right. \kern-0pt} {t_{x} }}}}{{\sum\nolimits_{k = 1}^{n} {{1 \mathord{\left/ {\vphantom {1 {t_{k} }}} \right. \kern-0pt} {t_{k} }}} }}$$

Therefore, the weight assigned to each experience is its *relative* recency—the recency of the experience relative to the summed recencies of all their experiences under consideration.

The TWR holds an advantage over models in parsimony by virtue of its lack of parameters. However, this approach provides no means to account for variation in how strongly recency influences the weighting of past experiences—which may be affected by a number of factors, such as overall rate of reinforcement (Mazur 1995) or volatility of reinforcement (Behrens et al. 2007). To address this limitation, the temporal weighting rule may be scaled with an exponential parameter (sTWR; Shahan and Craig 2017) (Eq. 4):

4 $$W_{x} = \frac{{{1 \mathord{\left/ {\vphantom {1 {t_{x}^{c} }}} \right. \kern-0pt} {t_{x}^{c} }}}}{{\sum\nolimits_{k = 1}^{n} {{1 \mathord{\left/ {\vphantom {1 {t_{k}^{c} }}} \right. \kern-0pt} {t_{k}^{c} }}} }}$$

Similar to the scaling used in a previous investigation of TWR (Devenport et al. 1997), this scalar term changes the degree to which recency influences weighting—or, in more functional terms, changes the relative steepness of the decay function for weights by raising recencies to the power of *c*. The current study incorporates *c* as a free parameter.

Regardless of whether a EWMA or the TWR is used to calculate values, the likelihood of an animal choosing a particular alternative is determined using a decision rule comparing the relevant values. For example, the matching law (Herrnstein 1961) suggests that the probability of an option’s selection matches the relative value of that option (Baum and Rachlin 1969); e.g., between two options, X and Y, the probability of an animal choosing option X corresponds to; *P*_*X*_ = *V*_*X*_/(*V*_*X*_ + *V*_*Y*_). This decision rule has been widely used in studies of dynamic averaging (Mazur 1995; 1996), specific evaluations of the TWR (Devenport and Devenport 1993; 1994), recent studies of behavioral allocation under changing conditions (Iigaya et al. 2019), and studies of choice in general (review in Houston et al. 2021).

Spontaneous recovery of choice is a poorly understood decision-making phenomenon that represents a challenge to many models of choice behavior and may offer a key differentiation in predictions between EWMA-based models and TWR/sTWR. SRC is analogous to the widely studied phenomenon of spontaneous recovery (SR), proper (Pavlov 1927; review in Rescorla 2004). In Pavlovian conditioning and single-response operant procedures, SR refers to the recurrence of an extinguished response following a delay between the end of extinction and a test of the association. A fundamental implication of SR is that the initial learning gained during training has not been entirely removed by the period of extinction, and that some change is occurring during the delay to cause the recurrence of responding. As such, SR has had various theoretical explanations, including the dissipation of inhibition during extinction (Pavlov 1927), replacement of stimulus elements within a conditioned set (Estes 1955), and differential retrieval of the initial learning resulting from time acting as a contextual cue (Brooks and Bouton 1993).

SRC, however, differs from SR in that the initial learning is a relative preference for one behavioral option over others, and the period in SRC that is analogous to “extinction” in SR may just be a period of different conditions—not necessarily a lack of reinforcement. The delay elicits the recovery of a previous preference between options, rather than an extinguished response. Two concurrent-schedules choice studies with pigeons (Mazur 1995; 1996) illustrate this effect empirically. The first study (Mazur 1995) comprised a series of experiments in which pigeons were trained to peck two keys, which were initially reinforced on equal concurrent variable interval (VI) schedules. After several daily 30-min sessions with equal schedules, one of the keys was assigned a richer schedule, and over the course of the sessions following this switch, subjects adjusted their behavioral allocation so that their response proportions asymptotically approached the new reinforcement proportions. However, at the beginning of these post-switch sessions, subjects’ response proportions initially reflected the reinforcement proportions of pre-switch sessions, rather than the proportions of the most recent session. In other words, following the 23.5 h inter-session interval, subjects partially recovered the preference they acquired during earlier sessions, rather than maintaining their preference from the end of the previous session.

A further investigation (Mazur 1996) followed a similar procedure, except switching one key to the richer schedule for only one, two, or three sessions before reverting to equal proportions of reinforcement. Again, subjects’ preferences at the beginning of sessions appeared to reflect earlier sessions, both during the switch to a richer schedule and the switch back to equal reinforcement proportions. Additionally, the number of richer-schedule sessions experienced was positively correlated with the strength of influence those sessions appeared to have on initial preferences in sessions after the reversion to equal reinforcement proportions. In one experiment, the introduction of a 3-day “rest period” was shown to attenuate the influence of past sessions when inserted between equal reinforcement proportions sessions and the sessions with a richer schedule for one key. This latter finding suggests a specific relationship between recency and strength of influence for past experiences.

Empirical study of SRC is limited, and primarily focused on testing the TWR’s account of the phenomenon. An early study with wild golden-mantled squirrels and least chipmunks studied preferences after a short and long delay (Devenport and Devenport 1994). For an initial training period, animals retrieved food from two feeding stands, one of which was baited with unhulled sunflower seeds, followed by an equally long period of training where the opposite stand was baited. Animals who experienced both trainings showed a shift in preference, where the more recently baited stand was exclusively preferred at the early test, and preference was roughly even between the two stands at the later test. In a further experiment, conditions of reinforcement were varied more frequently. Following a delay, subjects maintained their preferences for the stand that had previously been richer on average, rather than the stand that had been more recently baited (and subjects never chose a third stand that was never baited, suggesting the delay alone did not produce a reversion to an exploratory mode of foraging).

Similar results were obtained with an analogous procedure using dogs (Devenport and Devenport 1993) and later, with horses (Devenport et al. 2005)—subjects chose based on more recent information when available, but recovered their preference consistently with a regression to unweighted averages following the imposition of a temporal delay. In several studies examining spontaneous recovery of spatial preference, longer test delays were found to elicit spontaneous recovery in rats (Devenport 1998), mice (Lattal et al. 2003), and pigeons (Leising et al. 2015). In the two previously summarized studies with pigeons (Mazur 1995; 1996), the recovery of preferences following inter-session intervals was also consistent with a temporally weighted average. Additionally, the introduction of a three-day “rest period” in the latter study (1996) also produced changes qualitatively consistent with a temporally weighted average.

However, in comparison to the literature on SR (of a single response or association), the basic empirical properties of SRC, such as the relationship between test delay and recovery, have seen far less investigation. Understanding the relationship between test delay and SRC is highly relevant to our understanding of how animals value options over time for two reasons. First, if the passage of time alone effects a significant change in preference, it would suggest there is a fundamentally temporal dimension to animals’ valuation of options. Second, if the passage of time produces an increasing degree of recovery (like the negatively accelerated curves observed for SR, proper), the form of this function may provide insight into potential mechanisms of valuation, such as a temporally weighted average, that could underlie such a change in behavior. For SR, proper, the relation between delay length and extent of recovery follows a positive, negatively accelerated curve in various species and designs (e.g., Ellson 1938; Grant et al. 1958; Haberlandt et al. 1978; Quirk 2002; Robbins 1990).

The most robust exploration of the relationship between test delay and SRC occurs in a trial-based study with rats (Devenport et al. 1997). The study used a forced-choice training procedure where rats alternatingly sampled two options over two phases of equal length with differing conditions of reinforcement. Subjects were assigned to groups corresponding to four different test delays, which occurred before a test trial where subjects could freely choose between the two options, both unbaited. The shortest delay groups exclusively chose the most recently baited patch, but as delays increased, preference regressed to response proportions consistent with unweighted average patch values. Overall, these results show recovery consistent with a temporally weighted average and similar in form to studies of test delay and SR, proper. Similar results were obtained in a spatial memory task (Devenport 1998), where at shorter delays, subjects showed a preference for the more recently rewarded patch, but longer delays saw a regression to the unweighted average patch values.

TWR and EWMA-based models are both supported in different contexts, but the models crucially differ in how the weights assigned to past experiences change over time—which explains why they differ in their predictions for how preference might change over time. According to the TWR, because recency is calculated as 1/*t*_*x*_, the passage of time (increasing *t*) causes recency to decay hyperbolically. If time continues to pass without new experiences, *relative* differences in recency between experiences will shrink, and valuation will approach the unweighted average of reinforcement from those experiences. The explanation of SRC by the TWR is therefore relatively straightforward. If a period of reinforcement favoring option A is followed by a shorter period favoring option B, valuation just after the latter period will assign more weight to the experiences favoring option B—leading to a comparatively higher valuation of option B, and a low preference for option A. However, as time passes, and the relative difference in recency between the two periods becomes less significant, valuations will approach the unweighted averages of reinforcement from each option. Accordingly, the relative weight assigned to experiences favoring option B will decrease and the relative weight assigned to experiences favoring option A will increase—leading to a higher comparative valuation of option A and a corresponding recovery of preference for option A. By contrast, the weights functionally assigned by a EWMA model decay at the same exponential rate, preserving relative differences in value between options, regardless of the timepoint of valuation or whether the model increments with time or experience—meaning a EWMA alone will never predict SRC.

While the results reviewed tell a fairly consistent story that is more or less supportive of a temporally weighted average, it is important to note that they have been conducted over a relatively short timescale (the longest delay used was 48 h). However, both the TWR and models with an exponential decay of past experience are regularly applied to much longer timescales (e.g. Shahan and Craig 2017; Ranc et al. 2021). Additionally, many factors relevant to animal decision-making vary on the scale of weeks or months, such as seasonal changes in the availability of specific food sources or competition from migratory foragers. Studying these phenomena on a longer timescale allows for investigation into how foraging animals deal with slower-changing trends, as well as more ready application to human choice scenarios and behavioral treatments, which rarely are limited to a single day in duration.

The current study, therefore, aims to compare the performance of these models and determine if SRC actually occurs on these longer timescales. Although EWMA-based models will not predict SRC, it is possible that SRC does not occur at these longer timescales and a EWMA-based model might provide the best description of the data. Further, EWMA-based models merit inclusion in this comparison by virtue of their relative dominance in foraging, neuroscience, and artificial intelligence research (Lea and Dow 1984; Katahira 2015; Niv 2009; Zhang et al. 2013). The current study therefore directly compares the performance of a EWMA model, the TWR, and the sTWR in describing preference over a much longer timeframe (with delay intervals as long as 32 days) using a free-operant procedure analogous to the procedures that have produced SRC within a shorter timeframe.

## General methods

### Subjects

Experimentally naive male Long-Evans rats (approximately 72–92 days old) served as subjects. Rats were individually housed in a colony room controlled for humidity and temperature and illuminated on a 12:12 h light/dark cycle. Subjects were maintained at 80% of their free-feeding weight and provided access to water (ab libitum). Sessions were conducted at approximately the same time each day, 7 days/week.

### Apparatus

Ten identical operant chambers (30 × 24 × 21 cm; Med Associates) were housed in sound- and light-attenuating cubicles. Chambers included work panels on the front and back walls, with a clear Plexiglas ceiling, door, and wall opposite the door. On the back wall, a centered house light provided chamber illumination. On the front wall, two retractable levers were positioned on either side of a food magazine. Med-PC software controlled all experimental events and data collection.

### Procedure

#### Magazine and lever training

Experiments began with one session to train subjects to retrieve pellets delivered by a magazine. 30 pellets were delivered via magazine on a 60 s variable-time schedule, which illuminated for 3 s with each delivery. In four subsequent sessions, rats were trained on the lever press response. During these lever training sessions, the house light illuminated to signal the session’s beginning. Simultaneously, one lever extended and each lever press caused the lever to retract, the house light to extinguish, and a food pellet to be delivered. The delivery of a pellet initiated a 3 s consumption period, during which the magazine was illuminated, and the lever retracted. After this period, the same lever extended, and the house light was illuminated. For half of the subjects, the lever for option A extended during the first and third lever training sessions. For the remaining subjects, the lever for option B extended during the first and third sessions. The second and fourth sessions were used to train subjects on the opposite lever using the same method. Option A and B lever assignment was counterbalanced across subjects. Sessions terminated once 100 pellets were earned.

#### Phase 1 sessions

In Phase 1, daily sessions began with the house light illuminating and both levers extending. In the first session, the first lever press on either lever resulted in a pellet delivery. Following this first press, and for all other sessions during this phase, levers were baited on concurrent VI schedules. The option A lever was baited on a VI-10 s schedule while the option B lever was baited on a VI-90 s schedule. All VI schedules were constructed using ten intervals derived from the Fleshler and Hoffman (1962) distribution. To prevent animals from employing a simple alternating strategy, a 3 s changeover delay (as in Baum 1982) was employed (following each response, responses on the opposite lever did not produce pellet deliveries for a period of 3 s). Also, the delivery of a pellet initiated a 3 s consumption period, during which both levers retracted, and the session timer paused. Following this period, the levers extended again, and the timer resumed. Sessions terminated after 30 min. The number of daily sessions in Phase 1 varied between the three experiments (detailed in each experiment).

#### Phase 2 sessions

Beginning the day after Phase 1 ended, Phase 2 daily sessions continued in the same manner as Phase 1, but with reversed baiting conditions. That is, the option A lever was baited on a VI-90 s schedule while the option B lever was baited on a VI-10 s schedule. The changeover delay and 30-min duration remained the same. The number of daily sessions in Phase 2 also varied between the three experiments (detailed in each experiment).

#### Testing phase sessions

The testing phase comprised 1 or 2 test sessions for each group following various test delays (specific delays and tests detailed in each experiment). During test delays, animals remained in their home cage and maintained at their current weight. During test sessions, the house light illuminated to signal the beginning of the session, and both levers extended, but neither response was baited. The first 2 min of responding in each test session was used in data analysis to assess initial preference (responses on option A divided by total responses).

## Experiment 1

This experiment examined the effects of test delay on SRC by testing subjects at two different test delays to observe changes in relative preference following two phases of different reinforcement conditions. This within-subjects design followed the approach of previous studies of SRC (Devenport and Devenport 1993; 1994). The occurrence of a significant increase in the relative valuation of option A served as our criterion for evidence of SRC.

### Method

Ten rat subjects underwent magazine and lever training before the three-phase, free-operant serial reversal procedure detailed above (see General Methods). The length of Phase 1 (in which reinforcement proportions favored option A at a ratio of 9:1) and Phase 2 (in which reinforcement proportions favored option B at a ratio of 9:1) in this experiment was determined by simulating predictions for preference using the unscaled TWR model. Various lengths (including all whole numbers from 2 to 30 days) for both phases were simulated and a combination of phase lengths that maximized both the predicted reversal (during the phase of reversed conditions) and subsequent recovery of preference (following the test delay) was selected (Phase 1: 18 days; Phase 2: 7 days). Two tests were performed at two different test delays. Test 1 occurred on day 26, immediately after the final session of Phase 2 (a 1 day delay), and Test 2 occurred on day 50, after a delay of 25 days (equal to the summed lengths of Phases 1 and 2).

### Results

**Fig. 1:**
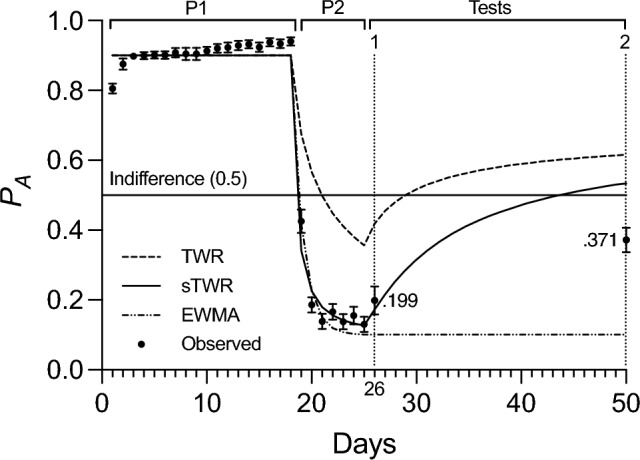
Preference for option A (*P*_*A*_) as a function of time (days) during Experiment 1. Phases 1 and 2 are indicated by P1 and P2, respectively, and tests are labeled above the data. Error bars represent the standard error of the mean. Predictions from the TWR, sTWR (*c* = 2.27), and a EWMA model (*β* = .603) are based on programmed reinforcement rates

Subjects acquired a preference for option A (*P*_*A*_) of 0.940 by the end of the first phase of 18 daily sessions, then reversed this preference (*P*_*A*_ = 0.131) by the end of the second phase of 7 daily sessions (Fig. 1). At Test 1, conducted on day 26, a low preference for option A was observed (*P*_*A*_ = 0.199). At Test 2, conducted on day 50, preference had increased to *P*_*A*_ = 0.372, but it did not rise above indifference between the two options (0.5). Because preference data was calculated as proportions, and therefore bounded between 0 and 1, the data were transformed to logits (log odds) to avoid violating assumptions of parametric statistical analysis (an overview of logits can be found in Cramer 2003). Logits are calculated simply by taking the natural log of the ratio of a proportion (*p*) to its complement (1-*p*). Proportions ranging from 0 to 1 will therefore range from negative to positive infinity as logits, better approximating a normal distribution of the data. The difference between preference in logits was statistically significant (*t*(9) = 5.81,* p* < 0.001). Three models were fitted to preference data throughout the experiment. Model predictions from the TWR, sTWR, and a EWMA model were calculated using the programmed rates of reinforcement. The Solver extension in Microsoft Excel was used to fit the sTWR and a EWMA model to the data, adjusting the parameters *c* and *β*, respectively, in minimizing residual sum of squares (employing the GRG-Non-linear algorithm). Observed data were better described by predictions from a fitted sTWR (*c* = 2.27, *R*^*2*^ = 0.982) than from TWR (*R*^*2*^ = 0.128) or from a fitted EWMA model (*β* = 0.603, *R*^*2*^ = 0.964). It is important to note that while the EWMA model provided a better fit overall than the TWR, it failed to predict the observed recovery, as expected. Information criterion comparison of these models favored the sTWR (*AIC* =  − 102.45, *BIC* =  − 101.56) over the EWMA model (*AIC* =  − 90.13, *BIC* =  − 89.24) with an evidence ratio of 8428:1 (see Klapes et al. 2018).

### Discussion

Behavior during Phases 1 and 2 approximated matching, with behavioral proportions roughly equal to reinforcement proportions by the end of each phase. In Phase 1, subjects acquired a strong preference for the richer alternative, option A, before gradually reversing this preference during Phase 2, where option B was the richer alternative. All three models (EWMA, TWR, and sTWR) accounted for this behavior well, as expected, but their performance differed for the two test sessions. The significant difference between the observed preference at Tests 1 and 2 provides compelling evidence of some SRC following the passage of time. However, these results do differ from the studies on which the experimental design was based (Devenport and Devenport 1993; 1994) in that preference appears to be retained, rather than reversed, at the 1 day delay—suggesting that the longer timescale of training does produce different results. This notion is further supported by the fact that late test performance was not described well by the TWR, as it was on a shorter timescale.

On its face, the occurrence of SRC acts as evidence against the account provided by a EWMA model of valuation, and potentially in support of an account by a weighted average like the TWR or sTWR. However, the result fell below the predictions of the TWR for this later test and was much better described by the sTWR (with a fitted parameter value of *c* = 2.27). The poor performance of the TWR in describing these data suggest that the parameter *c* is indeed necessary to account for differences in scaling (such as in timescale). Given the sTWR’s relative success in describing the data, we hypothesized that revising the lengths of the two initial phases to correspond with the predictions of the sTWR could produce more significant recovery of choice and incorporated this insight to the design of Experiment 2.

## Experiment 2

Experiment 2 followed the same basic design and procedure of Experiment 1, only changing the lengths of Phase 1 and Phase 2 based on the results of Experiment 1. All other aspects of the procedure were replicated, including within-subjects comparison.

### Method

Ten rat subjects again underwent magazine and lever training before the above-described serial reversal procedure. To determine phase lengths for Experiment 2, predictions for preference from the sTWR (with the parameter value that best fit the data from Experiment 1: *c* = 2.27) based on various lengths (including all whole numbers from 2 to 30 days) for both phases were simulated, and phase lengths (Phase 1: 14 days; Phase 2: 2 days) were selected by the same criteria as Experiment 1: maximizing the predicted reversal and subsequent recovery of preference.

### Results

**Fig. 2:**
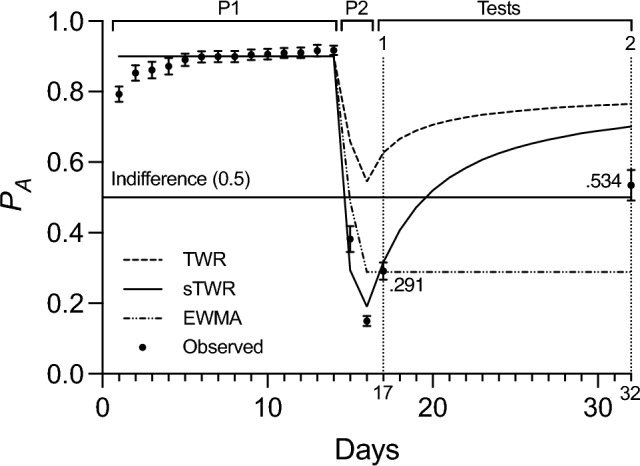
Preference for option A (*P*_*A*_) as a function of time (days) during Experiment 2. Phases 1 and 2 are indicated by P1 and P2, respectively, and tests are labeled above the data. Error bars represent the standard error of the mean. Predictions from the TWR, sTWR (*c* = 2.53), and a EWMA model (*β* = .514) are based on programmed reinforcement rates

Subjects again acquired a strong preference for option A (*P*_*A*_ = 0.917) by the end of the first phase, then reversed this preference for option A (*P*_*A*_ = 0.150) by the end of the second phase (Fig. 2). Test 1 was conducted the day after the second phase, and a low preference for option A was observed (*P*_*A*_ = 0.291). Test 2 was conducted after a test delay of 16 days from the end of the second phase, and although preference significantly increased to 0.534 (t-test of logit preference between tests: *t*(9) = 5.162, *p* < 0.001) from Test 1, it remained within a standard error of the mean (0.043) of indifference (0.5). Model predictions from the TWR, sTWR, and a EWMA model were calculated using the programmed rates of reinforcement and fit by the method described in Experiment 1. The observed data were again better described by a fitted sTWR (*c* = 2.53, *R*^*2*^ = 0.947) than the TWR (*R*^*2*^ = 0.044) or a fitted EWMA (*β* = 0.514, *R*^*2*^ = 0.895). The information criterion comparison favored the sTWR (*AIC* =  − 165.03, *BIC* =  − 163.73) over EWMA (*AIC* =  − 146.95, *BIC* =  − 145.66; evidence ratio of 473:1).

### Discussion

All three models again described Phases 1 and 2 well, but performance differed for the two test sessions. The significant difference between the observed preference at Tests 1 and 2 provided further evidence of SRC following the passage of time, and further support for the account of the sTWR over the account by a EWMA model of valuation. The results again differed from previous studies of SRC (Devenport and Devenport 1993; 1994), in that a 1 day delay was not sufficient to elicit SRC and the late test failed to show the same degree of recovery as in those shorter-timescale designs.

However, while the observed Test 1 preferences in both Experiments 1 and 2 fell within a standard error of the predicted preferences, observed preferences at Test 2 were considerably lower than predictions from the sTWR or TWR, and well below what would be expected based on previous studies of SRC (Devenport and Devenport 1993; 1994). This discrepancy, appearing in both experiments, suggests the possibility of a testing effect, with the experience of Test 1 potentially influencing performance during Test 2. During testing, subjects experienced a 30-min session during which previously reinforced alternatives are no longer reinforced. This experience, according the sTWR, should not have any effect on relative valuations between the two options—though it would decrease the absolute value of those options (Test 2 valuations and preference by sTWR without the extinction experience: *V*_*A*_ = 249.95, *V*_*B*_ = 150.05, *P*_*A*_ = 0.625; with the extinction experience: *V*_*A*_ = 198.00, *V*_*B*_ = 118.87, *P*_*A*_ = 0.625). For a EWMA, extinction experience after training has no effect on the decay of valuations, so including or excluding the preference makes no difference in the model’s predictions (Test 2 valuations and preference by sTWR with or without the extinction experience: *V*_*A*_ = 0.36, *V*_*B*_ = 0.89, *P*_*A*_ = 0.289). Indeed, in the studies that informed the design of experiments 1 and 2, SRC occurred at a late test despite the experience of extinction at an early test (Devenport and Devenport 1993; 1994). However, while the relative preference between the valuations of the two options may hypothetically have been preserved, this period of extinction could be affecting other factors relevant to performance of responses at the later test, especially on the longer timescale in use.

The notion that extinction conditions during a test might influence later testing is not novel. In a single operant response procedure (Skinner 1938), when two tests were performed under extinction conditions for the recovery of an extinguished response, the first test occurring the day after the response had been extinguished, the second test occurring 43 days later. For a group that experienced both tests, SR was significantly attenuated at the later test, relative to a group that was only tested at the later time. This difference (between the group that tested twice and the group only tested later) suggests that performance during the later test may have been influenced by the extinction conditions experienced during the first test. In a similar single-response procedure (Ellson 1938), recovery of a single bar-pressing response was tested after the response had been extinguished for four groups, each tested with a different delay: 5.5, 25, 65, or 185 min. The groups’ responding during those tests fell along a negatively accelerated curve, consistent with the predictions of valuation from a temporally weighted average like TWR. In short, we hypothesized that investigating recovery with a between-subjects design may isolate the effect of time delay from the effect of continued extinction, a hypothesis we then tested with the between-subjects design of Experiment 3.

## Experiment 3

Experiment 3 examined the relation between test delay and SR of choice using a between-subjects comparison of preference, exposing all subjects to the same history of reinforcement, but instituting a different test delay length for each of four groups. Because a testing effect could potentially have moderated the relationship between recovery and test delay in the within-subjects designs of Experiments 1 and 2, a between-subjects design was employed to provide a more robust test of the quantitative predictions of sTWR and a direct comparison of sTWR and a EWMA model for the relationship between test delay and recovery of choice.

### Method

Forty rat subjects underwent magazine and lever training before the above-described three-phase, free-operant serial reversal procedure. Phase 1 and 2 lengths were equivalent to those used in Experiment 2 (Phase 1: 14 days; Phase 2: 2 days). At the end of Phase 2, rats were assigned to 4 groups using a rank-match process based on the average preference during the last two days of phase 2, such that the 4 rats with the highest preference were assigned to separate groups, followed by the 4 rats with the next highest preference, and so on. Groups were further adjusted to ensure that their average preference did not significantly differ for last two days of Phases 1 or 2 (Phase 1 terminal logit preference comparison ANOVA: *F*(3,35) = 0.156, *p* = 0.931; Phase 2 terminal logit preference comparison ANOVA: *F*(3,35) = 0.089, *p* = 0.966). Group 1 was tested with a 1 day test delay (testing on day 17, the day after phase 2), Group 2 with a 3 day test delay (testing on day 19), Group 3 with an 8 day test delay (testing on day 24), and Group 4 with a 32 day test delay (testing on day 48). Delays were chosen based on simulated preference using the sTWR and the best fit parameter value from Experiment 2: the 1 day delay was chosen to maintain consistency with previous experiments; the remaining delays were chosen to maximize the difference in preference predicted at each time-point (each successive delay corresponded to a predicted increase of approximately 0.1. This approach was chosen over evenly distributing the tests over the test period because recovery was expected to follow the predictions of sTWR (as in Devenport et al. 1997), and an even distribution of tests would be associated with smaller predicted changes at the later tests.

### Results

#### Regression analysis

**Fig. 3:**
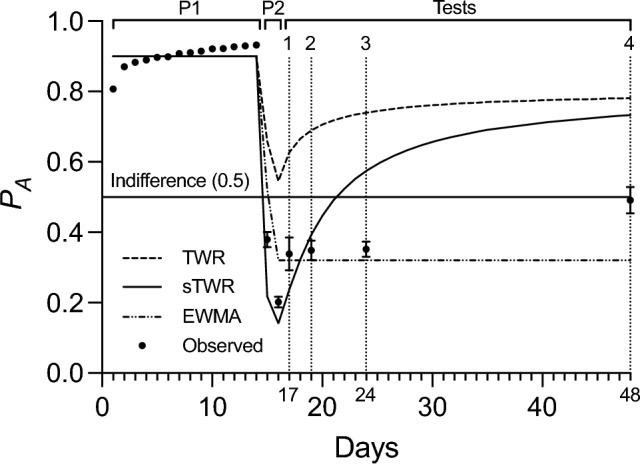
Preference for option A (*P*_*A*_) as a function of time (days) in Experiment 3. Phases 1 and 2 are indicated by P1 and P2, respectively, and tests are labeled above the data. Predictions from the sTWR use the best fit parameter value of *c* = 3.15. Predictions from a EWMA model use the best fit parameter value of *β* = .47. Test sessions occurred on days 17, 19, 24, and 48, corresponding to delays of 1, 3, 8, and 32 days, respectively

Observed preference data during the testing phase (Fig. 3), showed a monotonic increase as a function of test delay. However, the data failed to take the negatively accelerated curvilinear form found in previous studies of SRC (such as Devenport et al. 1997). While this discrepancy will be discussed and interpreted in greater detail below, visual analysis on the limited number of tests failed to suggest a specifically curvilinear form for the relationship. Given this uncertainty in form, a linear regression analysis was conducted to quantitatively assess the significance of the relationship between test delay and preference.

A linear regression fit of the data revealed a significant, positive relationship between delay and preference in logits (*p* = 0.004, *R*^*2*^ = 0.20). Test session data as proportions and logits can be found in Table 1, while the summary statistics of this linear regression fit can be found in Table 2. Because the animals received no new experience during the testing phase until their test session, the role of delay during this time as a significant positive predictor of preference constitutes strong evidence for SRC. However, it is notable that recovery never increased above indifference (indifference—a proportion of 0.5, transforms to a logit value of 0), similarly to the data gathered in Experiments 1 and 2.

**Table 1:** Experiment 3 test session data

Test delay	Day	Proportional Preference for A	*SE*	Logit Preference for A	*SE*
1	17	.339	.047	− 763	.261
3	19	.357	.034	− 620	.156
8	24	.359	.037	− 601	.159
32	48	.491	.038	− 040	.155

**Table 2:** Regression of test delay’s effect on logit preference in Experiment 3

	Estimate	*SE*	*95% CI*		*p*
			*LL*	*UL*	
Intercept	− 0.747	.121	− 0.992	− 0.502	< .001
Test Delay	0.022	.007	.007	.037	.004

#### Model comparison

Model predictions from TWR, sTWR, and a EWMA model were again calculated using the programmed rates of reinforcement to allow for unified predictions across all groups and fit by the method described in Experiment 1. Unlike in Experiments 1 and 2, sTWR failed to outperform the EWMA model in accounting for the data obtained. The EWMA model (*β* = 0.476, *R*^*2*^ = 0.940) showed a better fit than sTWR (*c* = 3.15, *R*^*2*^ = 0.876) or TWR (*R*^*2*^ = 0.158). Similarly, information criterion comparison favored the EWMA model (*AIC* =  − 108.71, *BIC* =  − 107.71) over sTWR (*AIC* =  − 94.09, *BIC* =  − 93.09), with a *ΔIC* of 14.61, constituting an evidence ratio of ~ 1488:1 in favor of the EWMA model.

### Discussion

Behavior during the test sessions was not well modeled by any of the three models. Preference during these sessions remained relatively constant for the first three tests, then increased to roughly approximate indifferent responding. Although our model comparison favored the EWMA model, this result is somewhat misleading. While it is true that the data are better fit by the EWMA model than TWR or sTWR over the course of the experiment, the test session data stand in stark disagreement with the EWMA model’s account of the effect of test delay. Since EWMA models of valuation do not predict changes in preference without new experience, the observed evidence of statistically significant SRC is fundamentally inconsistent with a EWMA-based account. Simultaneously, while the observed recovery is qualitatively consistent with an account from a weighted average like TWR or sTWR, these results are quantitatively inconsistent with the form and extent of SRC predicted by TWR or sTWR—to the point that the static preference predicted by the EWMA model is better supported by the comparison. In short, none of the three models under investigation were decisively more effective in describing the test session data.

Taking a step back, the conundrum of interpreting Experiment 3’s results might be best understood by examining three salient features of the test data in turn: 1) preference increased at the shortest test delay of 1 day; 2) preference did not increase for the intermediate test delays of 3 and 8 days; and 3) preference increased again at the longest test delay of 32 days. Since the account from the TWR could be provided by the sTWR (if the best fit value of *c* was 1), and this account was roundly outperformed in the model comparison, this examination will focus on contrasting the accounts of a EWMA model and the sTWR in explaining these three features—for clarity and simplicity.

The first feature, the increase from the end of Phase 2 to the first test (1 day delay), poses challenges for EWMA models, because preference at this first test falls well above what would be predicted by a EWMA model fit to just the data from Phase 1 and 2: the model (with a fitted *β* value of 0.476) predicts a preference of 0.199, while at the first test, subjects produced an actual preference of 0.339 (SEM = 0.047.). If one were to perform the same calculation using the sTWR, the model (with a fitted *c* value of 2.19) predicts a preference of 0.372. Therefore, this first feature of the data, at least on its face, seems to better support an sTWR account.

On the other hand, the second feature of the data, the lack of increase at test delays of 3 and 8 days, is inconsistent with the sTWR. Subsequent test session predictions from the sTWR (fit to Phase 1 and 2 alone, *c* = 2.19) rise above indifference (0.5), but the observed data for these groups shows no increase. In isolation, the lack of increase in preference from the 1-day delayed test to the 3- and 8-day delayed tests appears qualitatively consistent with a EWMA account—but all three of these tests represent increases in preference from the end of phase 2, inherently contradicting the predictions of a EWMA.

The third feature of the data, the increase seen at the final test (32 days), is difficult to explain by either model. From the perspective of a EWMA account, this late increase is inconsistent with a static account of preference, even if we ignored the increase from the end of Phase 2 to the first three tests. From the perspective of an sTWR account, this late increase is also unexpected, given that the sTWR would predict a negatively accelerated curve of recovery. We conclude that neither of these models in their current formulation describe the test data particularly well.

In addition, we found little evidence to suggest that the potential testing effect which shaped the design of Experiment 3 actually occurred—though the lack of evidence for such an effect is not conclusive evidence the effect was absent. Preference again failed to rise above indifference, as in Experiment 2, even at the latest test. Further, we conducted an additional test with all of the first three test groups on day 48 to compare their preference during a second test to the preference of the group only tested once and found no significant difference between groups with a one-way ANOVA of logit preference (*F*(3, 35) = 0.105,* p* = 0.957).

## General discussion

Together, the present experiments outline a consistent, but difficult-to-interpret finding: SRC can be reliably produced by time’s passage using the serial reversal procedure and the long test delays employed—but the form and extent of that recovery is poorly described by current approaches to modeling it. Further, these results are inconsistent with the most comparable previous study of the phenomenon (Devenport et al. 1997). In Experiments 1 and 2, exposing subjects to a longer test delay produced a significant increase in preference. While these experiments may have faced some confounders due to their within-subjects design, Experiment 3 similarly found a significant positive relationship between test delay and preference with a between-subjects design. However, at no point in any of the three experiments did recovery significantly rise above indifference (equal allocation between options), and the recovery obtained was poorly described by the models under investigation.

While these models’ failure in description may result from the inaccuracy of these approaches in describing the valuation of options—it could also result from an incorrect formulation of the decision rule employed for choosing between options. In essence, we must now ask if the failure of these models should be attributed to an inadequate account of valuation (modeling the process of integrating experience) or an inadequate account of choice (modeling the decision kernel guiding allocation based on calculated valuations).

Let us first turn to potential interpretations of these data that relate to the valuation functions employed. In all three experiments, we tested the performance of valuation models by evoking SRC. This effect, if we accept our current decision rule as accurate (for the moment), would provide evidence of a specific characteristic of how these functions calculate value: as experience fades into the past, its weight in decision-making decays at a decreasing rate. The weights assigned to past experience by the TWR or sTWR produce a hyperbolic decay of this form: experience loses weight more and more slowly as time goes on. By contrast, EWMA models functionally assign weight that decays at a constant exponential rate—meaning that relative preference should remain static without new experience. The fact that SRC occurred in all three experiments serves as evidence against a EWMA account. At the same time, in Experiment 3, which provided higher resolution of this recovery, the TWR and sTWR failed to predict the form of the data, and were actually outperformed quantitatively by the EWMA model, suggesting neither of the approaches under primary investigation were effective in modeling these data.

However, there is a class of valuation models that does relatively well in accounting for these data, with some caveats. Recent work in neuroscience has promoted the use of a multiple-timescale^1^ version of the EWMA model (Iigaya et al. 2019). The simplest version of such a model actually employs two EWMAs, a “fast” integrator with a larger *β* parameter (e.g., *β*_*x*_; corresponding to a shorter timescale), and a “slow” integrator with a smaller *β* parameter (e.g., *β*_*y*_; corresponding to a longer timescale). The model then divides weight between the two integrators with a third parameter (*W*_*x*_). In effect, this arrangement allows the weight of past experience to decay at a variable rate, approximating the same sort of declining decay rate found in the hyperbolic weightings of the sTWR or TWR.

As previously discussed, this declining decay rate in the weightings of past experience makes a 2-timescale EWMA model capable of describing preference reversals and SRC. Indeed, when a 2-timescale EWMA model is fit to data from Experiment 3, we find the best performance so far (*R*^*2*^ = 0.979; see Table 3 for full model comparison). Description can be improved even further with a 3 timescale EWMA model. This model, by employing three integrators, requires 3 learning rate parameters (e.g., *β*_*x*_, *β*_*y*_, *β*_*z*_) and 2 parameters to determine the weight assigned to each of the 3 integrators (*W*_*x*_, *W*_*y*_). The fit produced by this 3-timescale, 5-parameter EWMA model closely describes the data (*R*^*2*^ = 0.988).

**Table 3:** Experiment 3 model fit comparison

Model	Parameter fit	*RSS*	*R*^*2*^	*AIC*	*BIC*
TWR	*none*	0.643	.513	− 66.74	− 65.75
sTWR	*c* = 3.15	0.164	.876	− 94.09	− 93.09
sTWR (2 timescale)	*c*_*x*_ = 28.30 *c*_*y*_ = 1.23 *W*_*x*_ = .552	0.023	.981	− 129.61	− 126.62
EWMA	*β* = 0.476	0.079	.940	− 108.71	− 107.71
EWMA (2 timescale)	*β*_*x*_ = .823 *β*_y_ = .382 *W*_x_ = .630	0.028	.979	− 125.76	− 122.77
EWMA (3 timescale)	*β*_x_ = .921 *β*_y_ = .00053 *β*_z_ = .437 *W*_*x*_ = .483 *W*_*y*_ = .00000028	0.015	.988	− 133.54	− 128.56

However, our study represents the first application of these models to the effects of test delay on spontaneous recovery of choice—and the fits from these many-parameter models may be somewhat misleading. Behavior in Phase 1 and 2 of Experiment 3 approximates simple matching of behavior to reinforcement proportions,^2^ meaning that testing acts as the crucial period of performance for these models. With only 4 test sessions, models with more parameters are simply more capable of closely describing the data in post-hoc analysis. For example, we could take the same approach to the sTWR as the multi-integrator EWMA models, and use two valuations from separate sTWR integrators, each with their own *c* parameter and a parameter to determine weight between them. With this 2-integrator sTWR model (employing 3 parameters: *c*_*x*_, the scalar parameter of one integrator, *c*_*y*_, the scalar parameter of the other integrator, and* W*_*x*_, the parameter dictating the relative weight given to the first integrator), we can achieve better performance (*R*^*2*^ = 0.981) than the 2-integrator EWMA (which also has 3 parameters), and comparable performance to the 3-integrator EWMA (which has 5 parameters). While this 2-integrator version of the sTWR is too theoretically fraught and computationally intensive to seriously consider other than as a hypothetical, it serves to illustrate the potentially misleading success of the multi-integrator EWMA fits. So, while it is possible that these data are truly best *described* by the 3-integrator EWMA model, it is also possible that they may not be best *understood* by this account. As such, these models do merit discussion, but further empirical work is needed to validate their account of behavior under other circumstances.

To complicate things, there is another factor that could be affecting the learning rates of our valuation functions: volatility. Here, we define volatility specifically as the variance of reinforcement conditions in the environment. There is limited, but compelling evidence to suggest that volatility modulates learning rates, reflected in the learning rate parameter(s) of the EWMA models or the scalar parameter of the sTWR. There is evidence that humans optimally tune learning rates in response to volatility (Behrens et al. 2007). Choice behavior by monkey subjects has also been described well by a model which adjusted learning rate in response to volatility (Saito et al. 2014). Further, in a recent study with rats (Piet et al. 2017), subjects optimally adjusted their learning rate in response to volatility. Similarly, a recent model for learning based on the joint estimation of stochasticity and volatility (based on the idea that optimal decisions in more volatile conditions require higher learning rates) showed efficacy in simulating both human and animal data (Piray and Daw 2021). Our analysis employed learning rate parameters that remained constant over the course of the experiment, so future model development should explore the incorporation of learning rates that vary with volatility.

In summary, testing data from Experiment 3 challenges the accounts of two primary models under investigation, the sTWR and a EWMA. While these data are well-described by more complex models with more parameters, it is still unclear if those models are actually providing an explanation of recovery observed during testing, or if those models simply have more capability to fit to the test data. Further study will likely clarify this question, but before we can be confident that these data are best explained by investigating these functions, we should turn to an equally critical question: whether the formulation of our decision rule needs revision.

**Fig. 4:**
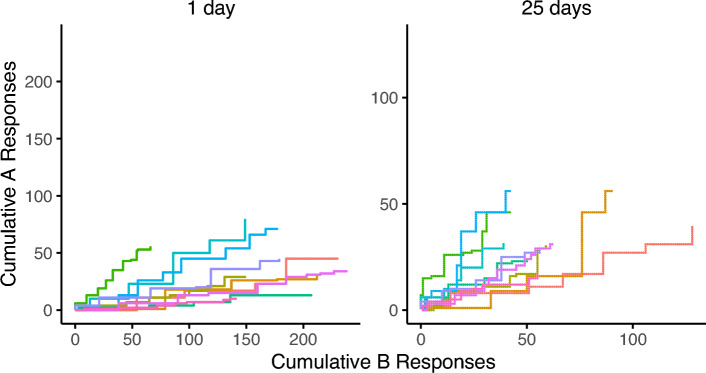
Cumulative responding toward options A and B during the first two minutes of test sessions in Experiment 1 by test delay. Panel titles describe the test delay, while color indicates individual subjects

**Fig. 5:**
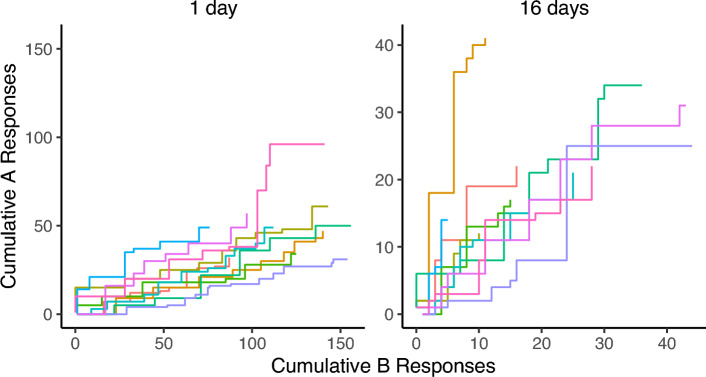
Cumulative responding toward options A and B during the first two minutes of test sessions in Experiment 2 by test delay. Panel titles describe the test delay, while color indicates individual subjects

**Fig. 6:**
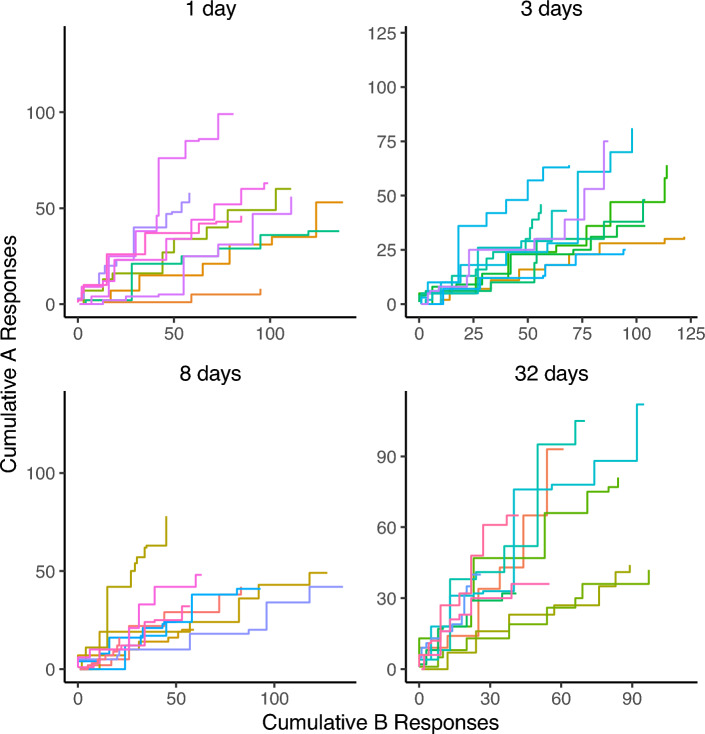
Cumulative responding toward options A and B during the first two minutes of test sessions in Experiment 3 by test delay. Panel titles describe the test delay, while color indicates individual subjects

The second theoretical locus of potential responsibility for the form of recovery obtained in these experiments is our decision rule. In all three experiments, at the longer (or longest) delay tested, recovery of choice failed to rise significantly above indifference (0.5). Following Experiments 1 and 2, we suspected that a testing effect of some sort may have influenced our data and attenuating recovery—but the between-groups comparison of Experiment 3 failed to show a greater degree of recovery. Together, these three experiments might suggest a potential drift toward indifference (or exploration) as test delay increases, possibly because subjects are reverting to a more exploratory or stochastic mode of behavior. Such a drift does not appear to be an acute effect of the extinction experience during testing, as the trends of preference observed in each test remain relatively consistent over the first two minutes of the test session (Figs. 4, 5, and 6). Setting aside valuation models for the moment, we will explore how different formulations of our decision rule could produce such a drift.

The decision rule we employed was based on an extension of the matching law (Herrnstein 1961). The matching law has been widely studied in diverse choice scenarios with diverse subjects and experimental methods (for a review of empirical study, see Davison and McCarthy 1988). The formulation we employed follows the “concatenated” matching law (Baum and Rachlin 1969)—which posits that the construct of value could comprise the product of various parameters for options (like reinforcement rate, magnitude, or immediacy).

While we employed a proportional formulation of the matching law, the drift to indifference may be understood better using the ratio formulation of the generalized matching law (Baum 1974). This version of the matching law set the ratio of behavioral allocation (*B*_*1*_*/B*_*2*_) as equal to the ratio of reinforcement (*R*_*1*_*/R*_*2*_), which is multiplied by a parameter corresponding to bias (*b*) between options and raised to a parameter (*s*) representing sensitivity to reinforcement (*B*_*1*_*/B*_*2*_ = *b*(*R*_*1*_*/R*_*2*_)^*s*^). Within this theoretical framework, a drift to indifference could represent a decrease in sensitivity: as sensitivity decreases, the ratio of reinforcers will approach unity, which here equates to equal allocation to the two options, or indifference. By this account, animals could show indifferent responding (equally allocating to both options) despite preserving relative valuations. In other words, the animals could be tracking valuations by some process like we have described, but the ratio of those valuations may not be the prevailing factor controlling behavioral allocation.

Sensitivity has been theoretically linked to discriminability, following the notion that matching sensitivity to reinforcement ratios may be better conceptualized as the discriminability of reinforcer contingencies (Davison and Jenkins 1985; Davison and Nevin 1999). With this lens, we may view this drift as a decrease in the discriminability of either the two options themselves (stimulus–response discriminability: d_sb_, i.e., the extent to which an animal can determine “which response is associated with this stimulus?”) or discriminability of the reinforcement schedules associated with the two options (response-reinforcement discriminability: d_br_, i.e., the extent to which an animal can determine “what is the reinforcement history associated with this response?”).

A change in either dimension of discriminability could occur with a variety of changes that occur during the testing phase. During the test delay, the options are not available to the subjects in their home cages, and when the options are finally made available, they are not baited. The dramatic drop in overall reinforcer rate during this time could have some influence on discriminability, as is suggested by the finding that rats shifted their responding to reflect the current distribution of reinforcement more slowly with lower overall rates of reinforcement (Bizo and White 1994). A decrease in discriminability could also simply result from the passage of time (White 2002), potentially due to psychophysical properties of the remembering process.

In contrast to this decision rule from matching theory, many explore/exploit models hypothesize that a softmax function guides the decision to exploit (choose the option with the highest value) or explore (choose another option) (Daw et al. 2006)—and the influence of the highest valued option is determined by an inverse temperature or “softmax gain” parameter (Addicott et al. 2017). In the context of Experiments 1, 2, and 3, the time delay could be somehow linked to a decrease in this temperature parameter, meaning the observed trend toward indifference could be seen as a strategic adaptation—reverting to a mode of exploring both options, despite preserving differences^3^ in valuation between the options. As a caveat, this interpretation would be somewhat inconsistent with previous findings where after a long delay, despite showing indifference between two previously reinforced patches, subjects almost never chose a third patch that was never reinforced or extinguished—suggesting that “exploration” would at least be limited only to previously reinforced patches. (Devenport and Devenport 1993; Devenport et al. 2005). Further analysis will be needed to evaluate the efficacy of this alternative.

Finally, while there is limited evidence showing the effect of volatility on variable learning rates in our valuation functions, it is also possible that volatility might influence the sensitivity/discriminability or inverse temperature of our decision rule. This notion follows the idea that when animals are in highly variable environments, there is an adaptive value in tending toward exploration (or indifference), which would entail more sampling of the various options to determine their uncertain value. In agreement with this idea, there is some evidence linking volatility to greater exploration, as quantified by a lower inverse temperature parameter (Knox et al. 2011; Wang et al. 2023). It is unclear whether volatility itself would produce an effect on discriminability, or whether volatility may simply moderate the effects of test delay or reinforcement rate on discriminability.

In support of this potential effect, there is some evidence that exposure to variable reinforcement conditions decreases sensitivity over time in a steady-state experiment (Todorov et al. 1983) and over the course of a series of experimental blocks with different reinforcement conditions (McLean et al. 2018)—but volatility’s effect on sensitivity for various lengths of test delay is essentially unexplored. Additionally, in several of the studies that examined the effect of volatility on valuation (Behrens et al. 2007; Saito et al. 2014; Piet et al. 2017), it is possible that the appearance of some changes in learning rates could also be produced by variation of the sensitivity of the decision rule employed, but further study is needed to explore this directly.

Similarly, further investigation may determine the potential influence of reinforcement rate and recency on sensitivity or discriminability. Additional study may also reveal whether volatility’s effect is best conceptualized as an influence on learning rate or an influence on the decision rule, but experimentally isolating and theoretically incorporating the effect of volatility could help to greatly improve our understanding moving forward. Finally, it is possible that the findings of these experiments may differ from previous work (i.e. Devenport et al. 1997) because they occur on a much longer timescale. To that end, future study could evaluate the performance of these models on various timescales to determine if circadian patterns influence dynamic averaging processes for rats, as has been found in honeybees (Cheng 2012).

## Data Availability

Data and other study materials are available upon request from the corresponding author.

## Footnotes

1 This multi-scale approach resembles the composite Brownian distribution of search segments used by ants in search behavior, which comprise two exponential distributions at different scales (Schultheiss et al. 2015).

2 It is interesting to note that overmatching (behavioral allocation that is more extreme than reinforcement proportions) was observed during phase 1 of all three experiments, despite consistent evidence that concurrent VI-VI schedules typically produce undermatching (Baum 1979) and overmatching has usually been observed only when switching options incurred significant travel requirements or change-over delays (Baum 1982). Given that the changeover delay employed was relatively short at 3 s, the cause of this overmatching is unclear.

3 Note that decision rules based on the difference between option valuations, such as the softmax functions employed in many explore/exploit models, have poorer empirical support than models employing decision rules based on the ratio of these valuations (Worthy 2008; Gibbon 1977).
